# Supplementary material for: Information content differentiates enhancers from silencers in mouse photoreceptors
Source: eLife. 2021 Sep 6;10:e67403. doi: 10.7554/eLife.67403 (PMC8492058; doi:10.7554/eLife.67403)
Supplement: Transparent reporting form [file elife-67403-transrepform1.docx]

***eLife’s* transparent reporting form**

We encourage authors to provide detailed information *within their submission* to facilitate the interpretation and replication of experiments. Authors can upload supporting documentation to indicate the use of appropriate reporting guidelines for health-related research (see [EQUATOR Network](http://www.equator-network.org/%20)), life science research (see the [BioSharing Information Resource](https://biosharing.org/)), or the [ARRIVE guidelines](http://www.plosbiology.org/article/info:doi/10.1371/journal.pbio.1000412) for reporting work involving animal research. Where applicable, authors should refer to any relevant reporting standards documents in this form.

If you have any questions, please consult our Journal Policies and/or contact us: [editorial@elifesciences.org](mailto:editorial@elifesciences.org).

**Sample-size estimation**

- You should state whether an appropriate sample size was computed when the study was being designed
- You should state the statistical method of sample size computation and any required assumptions
- If no explicit power analysis was used, you should describe how you decided what sample (replicate) size (number) to use

Please outline where this information can be found within the submission (e.g., sections or figure legends), or explain why this information doesn’t apply to your submission:

A prior power analysis is not applicable to our submission. Our prior exploratory work with MPRAs in the retina has established that three biological replicates is sufficient to reproducibly detect the effect sizes we discuss in the study. In these experiments, an appropriate sample size is determined by the reproducibility of biological replicates. Three replicates is the minimum number of replicates required to determine if a replicate is an outlier and is sufficient to detect small effect sizes given the size of the libraries and the depth of sequencing.

**Replicates**

- You should report how often each experiment was performed
- You should include a definition of biological versus technical replication
- The data obtained should be provided and sufficient information should be provided to indicate the number of independent biological and/or technical replicates
- If you encountered any outliers, you should describe how these were handled
- Criteria for exclusion/inclusion of data should be clearly stated
- High-throughput sequence data should be uploaded before submission, with a private link for reviewers provided (these are available from both GEO and ArrayExpress)

Please outline where this information can be found within the submission (e.g., sections or figure legends), or explain why this information doesn’t apply to your submission:

As indicated in “Materials and Methods - Retinal Explant Electroporation”, each experiment (2 libraries x 2 promoters) was performed in three biological replicates. A biological replicate is defined as a pool of five electroporated retinas. No data was excluded because of the high reproducibility between replicates. Sequencing data is available on GEO as indicated in “Code and Data Availability”.

**Statistical reporting**

- Statistical analysis methods should be described and justified
- Raw data should be presented in figures whenever informative to do so (typically when N per group is less than 10)
- For each experiment, you should identify the statistical tests used, exact values of N, definitions of center, methods of multiple test correction, and dispersion and precision measures (e.g., mean, median, SD, SEM, confidence intervals; and, for the major substantive results, a measure of effect size (e.g., Pearson's r, Cohen's d)
- Report exact p-values wherever possible alongside the summary statistics and 95% confidence intervals. These should be reported for all key questions and not only when the p-value is less than 0.05.

Please outline where this information can be found within the submission (e.g., sections or figure legends), or explain why this information doesn’t apply to your submission:

All details on data processing and statistical methods are found in the “Data Processing” and “Assignment of Activity Classes” sections of the Materials and Methods. Horizontal lines in violin plots represents median. Exact p-values, statistical tests, and N are always stated in the text and/or figures at each point where new results are described.

(For large datasets, or papers with a very large number of statistical tests, you may upload a single table file with tests, Ns, etc., with reference to sections in the manuscript.)

**Group allocation**

- Indicate how samples were allocated into experimental groups (in the case of clinical studies, please specify allocation to treatment method); if randomization was used, please also state if restricted randomization was applied
- Indicate if masking was used during group allocation, data collection and/or data analysis

Please outline where this information can be found within the submission (e.g., sections or figure legends), or explain why this information doesn’t apply to your submission:

This is not relevant to the study. There are no groups to be allocated in this work.

**Additional data files (“source data”)**

- We encourage you to upload relevant additional data files, such as numerical data that are represented as a graph in a figure, or as a summary table
- Where provided, these should be in the most useful format, and they can be uploaded as “Source data” files linked to a main figure or table
- Include model definition files including the full list of parameters used
- Include code used for data analysis (e.g., R, MatLab)
- Avoid stating that data files are “available upon request”

Please indicate the figures or tables for which source data files have been provided:

All data are provided as Supplementary Files and are referenced throughout the manuscript. Complete details of the Supplementary Files are in the section “Supplementary Files”. All code is available on Github as indicated in “Data and Code Availability”.
